# Quantitative Measurement of Cerebral Oxygen Extraction Fraction Using MRI in Patients with MELAS

**DOI:** 10.1371/journal.pone.0079859

**Published:** 2013-11-08

**Authors:** Lei Yu, Sheng Xie, Jiangxi Xiao, Zhaoxia Wang, Xiaodong Zhang

**Affiliations:** 1 Department of Radiology, Peking University First Hospital, BeiJing, China; 2 Department of Radiology, China-Japan Friendship Hospital, BeiJing, China; 3 Department of Neurology, Peking University First Hospital, BeiJing, China; The University of Chicago, United States of America

## Abstract

**Objective:**

To quantify the cerebral OEF at different phases of stroke-like episodes in patients with mitochondrial myopathy, encephalopathy, lactic acidosis, and stroke-like episodes (MELAS) by using MRI.

**Methods:**

We recruited 32 patients with MELAS confirmed by gene analysis. Conventional MRI scanning, as well as functional MRI including arterial spin labeling and oxygen extraction fraction imaging, was undertaken to obtain the pathological and metabolic information of the brains at different stages of stroke-like episodes in patients. A total of 16 MRI examinations at the acute and subacute phase and 19 examinations at the interictal phase were performed. In addition, 24 healthy volunteers were recruited for control subjects. Six regions of interest were placed in the anterior, middle, and posterior parts of the bilateral hemispheres to measure the OEF of the brain or the lesions.

**Results:**

OEF was reduced significantly in brains of patients at both the acute and subacute phase (0.266 ± 0.026) and at the interictal phase (0.295 ± 0.009), compared with normal controls (0.316 ± 0.025). In the brains at the acute and subacute phase of the episode, 13 ROIs were prescribed on the stroke-like lesions, which showed decreased OEF compared with the contralateral spared brain regions. Increased blood flow was revealed in the stroke-like lesions at the acute and subacute phase, which was confined to the lesions.

**Conclusion:**

MRI can quantitatively show changes in OEF at different phases of stroke-like episodes. The utilization of oxygen in the brain seems to be reduced more severely after the onset of episodes in MELAS, especially for those brain tissues involved in the episodes.

## Introduction

Mitochondrial myopathy, encephalopathy, lactic acidosis, and stroke-like episodes (MELAS) is a common type of mitochondrial disorder, characterized by neurological remissions and relapses, associated with progressive neuro-cognitive deficits [1]. Patients present with severe symptoms such as hemiparesis, altered consciousness, vision abnormalities after the acute onsets of stroke-like episodes. These symptoms may gradually resolve at the subacute phase. The stroke-like episodes are often followed by a complete recovery at the interictal phase. A mitochondrial DNA point mutation is most often the underlying genetic factor of the disease, which causes a failure of mitochondrial protein synthesis resulting in impaired ATP production. For example, 80% of MELAS cases are associated with the mutation of mitochondrial DNA A3243G, and various other mutations have been reported [2]. 

In contrast to the rapid progress in understanding the molecular pathophysiology of MELAS, the exact mechanism of the stroke-like episodes has not been fully elucidated. To clarify the mechanisms of MELAS, various pathophysiological parameters of the cerebral lesions *in vivo* have been assessed by neuroimaging, including cerebral blood flow, oxygen consumption, glucose metabolism, and oxidative stress. One of the important parameters defining oxygen consumption is oxygen extraction fraction (OEF) – the percent of the oxygen removed from the blood by tissue during its passage through the capillary network, which is comparatively stable in different cortices [3].

Because of the abnormalities of mitochondrial function, the defect in the oxidative metabolic pathways of energy production would decrease the cerebral oxygen utilization, thus decreasing the OEF. The quantification of OEF in patients can reflect the functional status of cerebral mitochondria. Previous measurements conducted by PET imaging techniques have demonstrated that cerebral OEF of patients with MELAS is generally decreased after the stroke-like episodes, especially in the affected lobes [4-6]. However, PET requires radioactive isotopes, and the required onsite cyclotron has limited its use in clinical care and clinical research. In light of recent advances in MR imaging, the discovery of blood oxygen level-dependent (BOLD) signal has allowed development of magnetic resonance imaging (MRI) methods targeted toward quantitative OEF imaging [7-9]. A new MR sequence, termed the gradient-echo sampling of spin echo (GESSE), was successfully developed to enable quantitative assessment of the OEF in brain tissue. The GESSE has been used to evaluate the misery perfusion in patients with brain ischemia and obtain reliable results of cerebral OEF [10]. In this study, we applied this promising method to investigate the cerebral OEF in patients with MELAS at different phases. The aim of our study was to investigate the change of oxygen metabolism in the brain tissues throughout the stroke-like episodes.

## Materials and Methods

The case-control study was approved by the Ethics Committee of Peking University First Hospital, Beijing, China. Written informed consent of participation in this study was obtained from every patient. In the case of pediatric patients, written informed consents were obtained from their parents. Patients participating in the study were recruited from the Department of Neurology of Peking University First Hospital. The diagnosis of MELAS was based on the presence of ragged red fibers in a muscle biopsy sample or the presence of mtDNA mutation besides the clinical symptoms. From December 2009 to December 2011, 32 patients at different phases were studied, and 35 MRI examinations were obtained. Three patients were examined twice at different phases. According to the time relationship to the stroke-like episodes, phases of MELAS were defined as following: (1) acute phase, within 10 days after the onset of stroke-like episode; (2) subacute phase, between 11 days and 1 month after the onset; and (3) interictal phase, at least 1 month after the stroke-like episodes. The MR examinations of patients were divided into 2 groups: group A consisted of 16 MR examinations at the acute and subacute phases (9 males, 7 females; mean age, 17 years; age range, 5–35 years); group B consisted of 19 MR examinations at the interictal phase (11 males, 8 females; mean age, 26 years; age range, 6–39 years). Abnormal high signal on T2WI and tissue swelling were detected in the brain lesions of patients in group A. In group B, the patients’ brains may show signs of atrophy and/or chronic lesions. Twenty-four volunteers (12 males, 12 females; mean age, 25 years; age range, 23–27 years) were recruited as the group C, which was the control group. The controls had no headache, epilepsy, head trauma, or other mental problems. No abnormalities were found on their brain MRI examinations.

**Figure 1:**
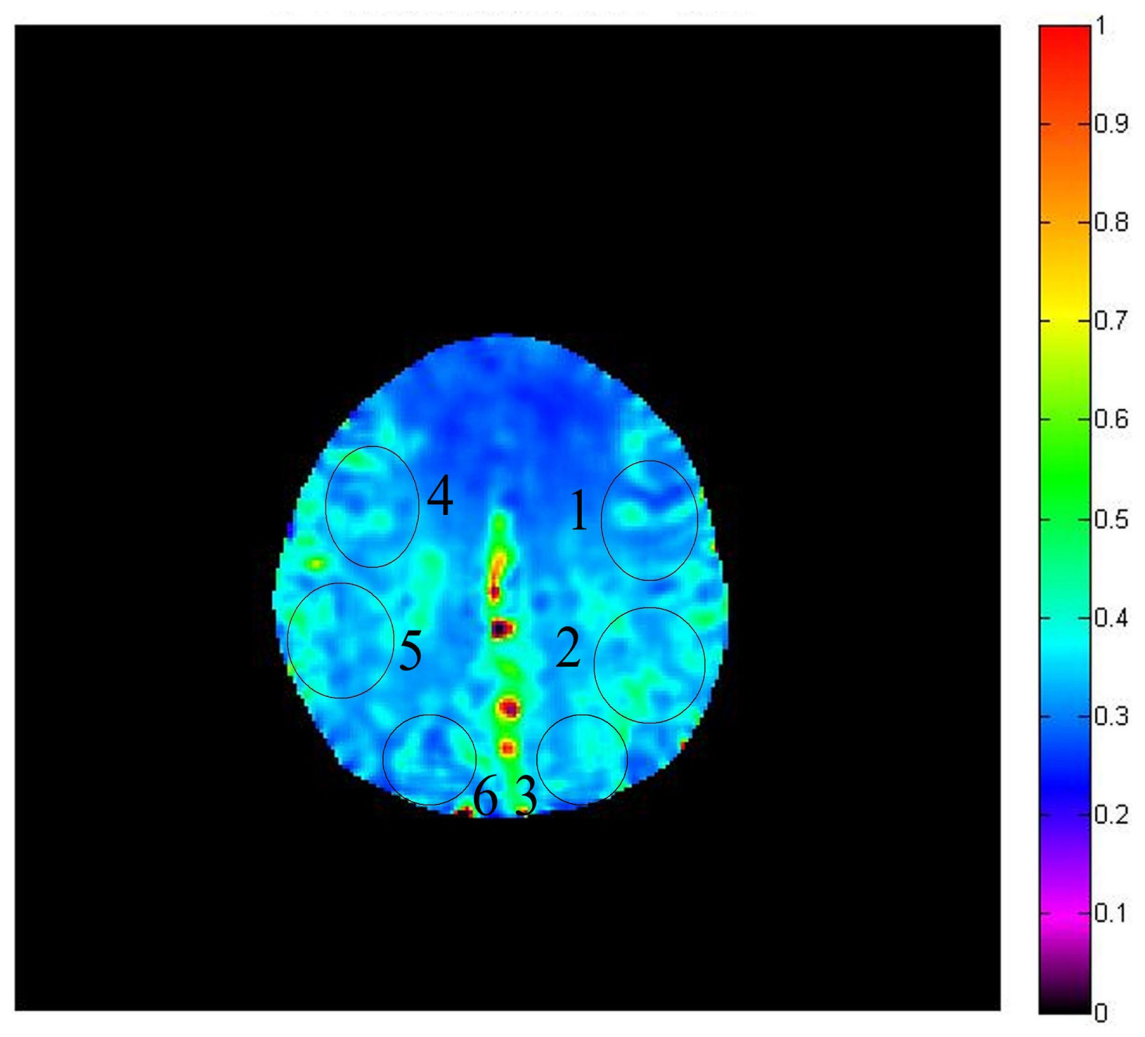
Example of the OEF map from a volunteer and locations of ROIs for the measurement of OEF. All data were expressed as mean ± SD. Kruskal-Wallis Test was used for comparing the difference of the cerebral OEF among 3 groups. Comparison of cerebral OEF between the affected brain regions and the contralateral hemispheres was conducted with Wilcoxon Signed Ranks Test. A value of *P*<0.05 was considered statistically significant.

The MRI examinations were performed on a 3.0-T whole-body MR scanner (Signa Excite TM; GE medical systems, Milwaukee, WI, USA) with an 8-channel head coil. The routine images obtained includedT1-weighted axial images, T2-weighted axial images, fluid attenuated inversion recovery axial images, and diffusion-weighted axial images. Arterial spin labeling (ASL) and GESSE sequences were then performed to obtain the hemodynamic information. ASL can be used to measure cerebral blood flow (CBF) by using intravascular water as the endogenous contrast agent. The parameters for ASL were: TR=800 ms, TE= 22.8 ms, EC=250 kHz, matrix=128×96, NEX=1, slice thickness= 6.0 mm, and space between the slices=1.5 mm. CBF maps were generated on the workstation by processing the ASL data. GESSE was performed to obtain the OEF imaging with the following parameters: TR=1.5 s, TE=56 ms, bandwidth=62.5 kHz, matrix=128×128, FOV=240×240 mm, section thickness=7.5 mm, NEX=4, scanning time=12 min 54 s. The section was located above the corpus callosum. We chose this section because bone-gas interface artifacts could be minimized and most of the lesions involved the brain on this section 32 images were obtained to be used as raw data, which were processed using the software developed in-house. To minimize effects of large background magnetic field inhomogeneities, which usually resulted in geometric distortion in the spin-echo images and severe signal-intensity loss in the gradient-echo images, software in the analysis automatically excluded voxels with low signal intensity–to-noise ratios resulting from artifacts and marked them as black regions. After OEF mapping was generated, six regions of interest (ROI) were placed from the left anterior to right posterior parts of the bilateral hemispheres to obtain the OEF of different ROIs, and the sizes of ROI were similar, covering both the gray matter and adjacent white matter. Figure 1 denotes the position of the ROIs for the measurement on the OEF map. While the artifacts areas were avoided, a radiologist slightly adjusted the location and size of some ROIs. We obtained six OEF values and then calculated the mean value, which was defined as the cerebral OEF of this section. The OEF map and the six ROIs are shown in Figure 1. OEF values of controls (mean ± SD) were obtained from healthy volunteers. 

## Results

**Figure 2:**
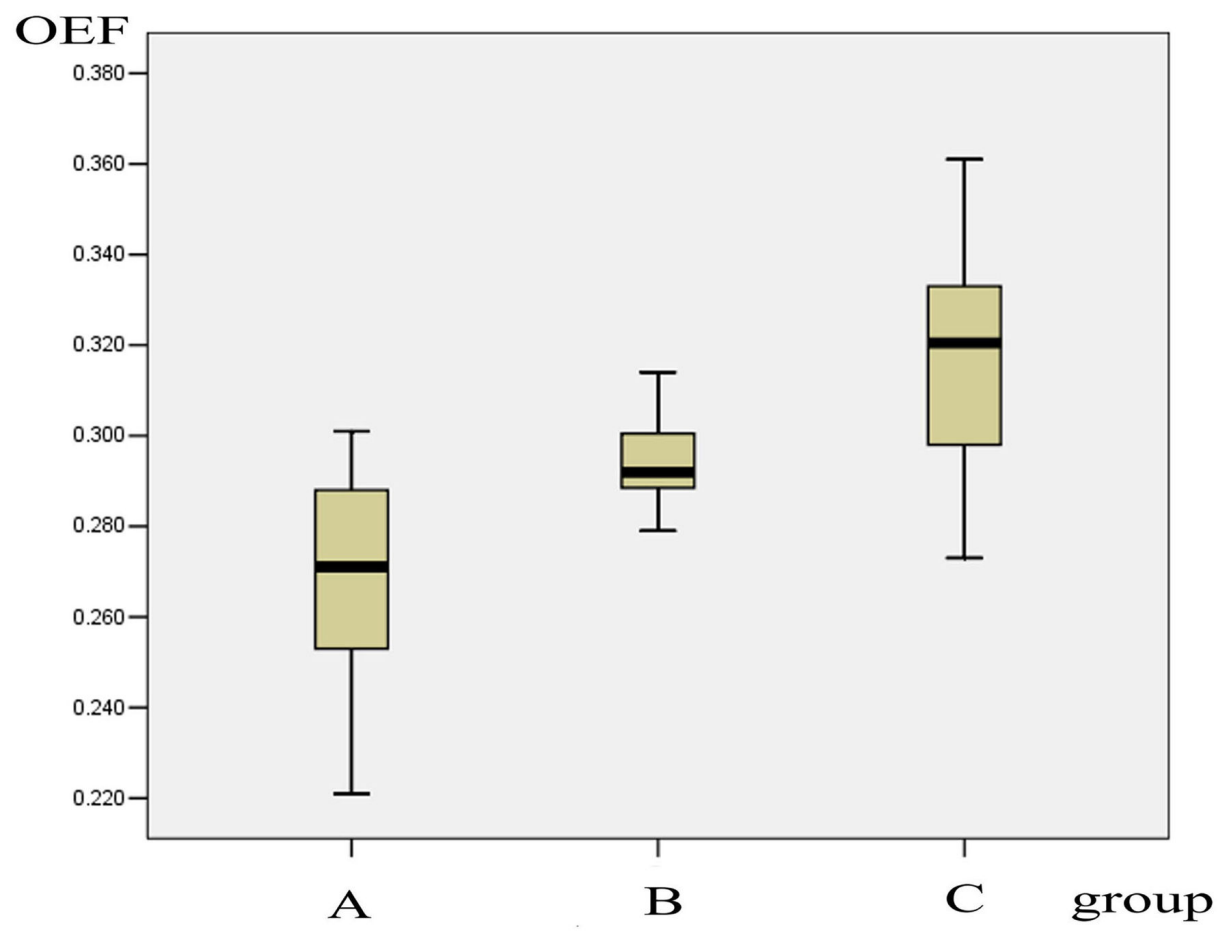
Graph shows the cerebral OEF of group A, B, and C. Whiskers mark the 10th and 90th percentiles, and boxes are bounded by the 25th and 75th percentiles.

Analysis of variance showed there was no difference among the various regions in the brains of control subjects. Thus, the cerebral OEF values of all ROIs in the controls were pooled to define the normal range of cerebral OEF, which was 0.316±0.025 with a 95% confidence interval of 0.306-0.327. The cerebral OEF values were 0.266±0.026 in group A and 0.295±0.009 in group B, which were significantly decreased compared with the control group (X^2^=25.800, *P*<0.001). Mann-Whitney Tests revealed significantly reduced cerebral OEF values at the acute and subacute phase relative to OEF at the interictal phase (*Z*=-3.446, *P*=0.001, two-tailed), as well as the interictal phase relative to the controls (*Z*=-2.850, *P*=0.004, two-tailed) . Figure 2 shows the bar graph of the OEF values of the three groups.

On the OEF maps of group A, 18 out of 96 ROIs were placed on the stroke-like lesions. The lesions included one in ROI 2, 11 in ROI 3, 2 in ROI 5, and 4 in ROI 6. The mean OEF value of these lesions was 0.259±0.045. The other 78 ROIs were uninvolved in the stroke-like episodes, and the mean OEF value was 0.268±0.033. Thirteen of the 16 patients at the acute and subacute phase had unilateral involvement in the cerebrum. When compared with OEF of contralateral uninvolved ROIs, these ROIs with acute and subacute lesions had significantly decreased OEF values by using Wilcoxon Signed Ranks Test (*Z*= -2.271, *P*=0.023). 

In contrast to the decreased cerebral OEF in the brains at the acute and subacute phase, increased blood flow was revealed in the stroke-like lesions on CBF maps. However, the extent of increased CBF was not consistent with that of decreased OEF. Increased CBF was always confined to the lesion region, while decreased OEF involved more extensive brain regions. At the interictal phase, the stroke-like lesions became chronic and exhibited decreased CBF.

**Figure 3:**
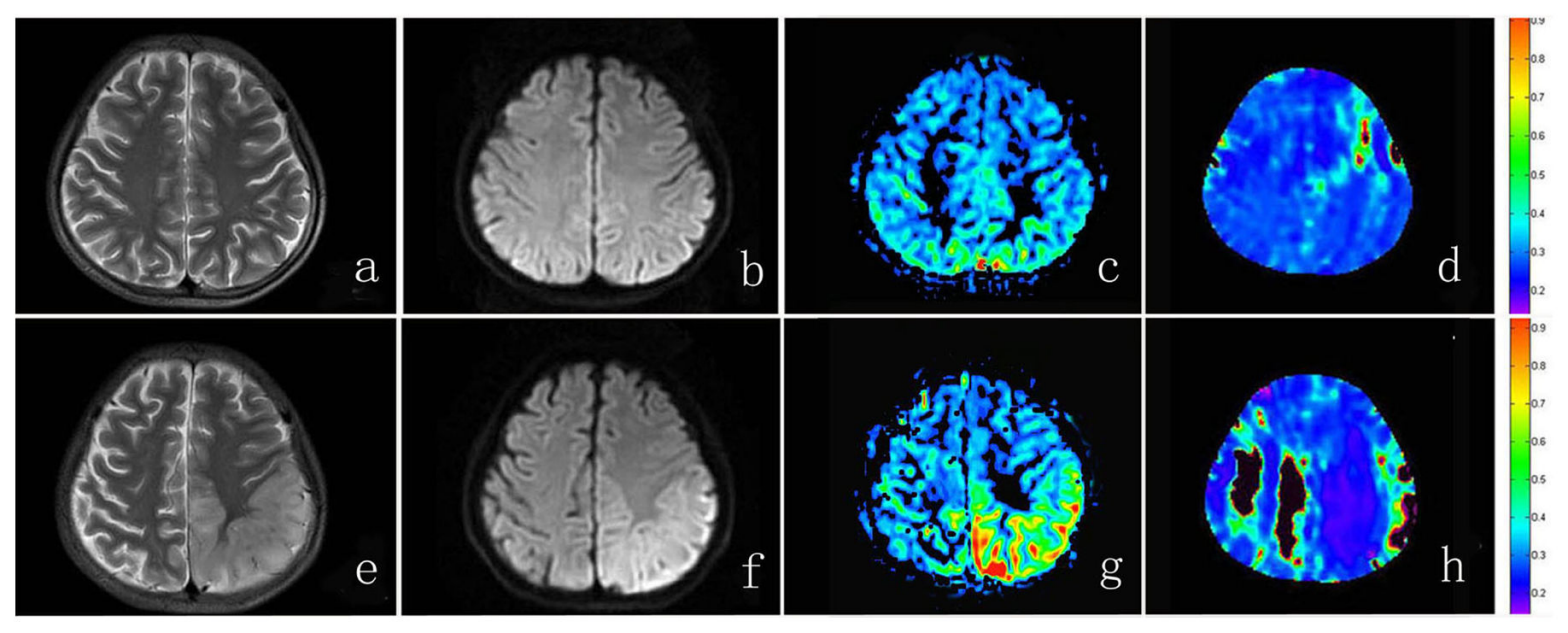
MRI in a 14-year-old boy with MELAS. The top row shows images at the interictal phase. T2WI image (A) , DWI image (B) and CBF map (C) are unremarkable. OEF map (D) shows global decreased OEF value relative to controls. The bottom row shows images at the acute phase, 9 days after the stroke-like episode. High signal and swelling of left parietal lobe can be seen on T2WI image (E) and DWI image (F). Increased CBF is depicted in the lesion on the CBF map (G). The OEF is markedly decreased across the brain, especially in the lesion region (H). The regions with low SNR are marked as black to be excluded from measurement.

Figure 3 depicts a case of MELAS at the interictal phase and at the acute phase. Compared with controls, the cerebral OEF was extensively decreased across the brain at the interictal phase in the patient. When the episode attacked, the lesion region showed markedly increased CBF but more reduced OEF relative to the interictal phase.

## Discussion

We found decreased cerebral OEF in the brains of patients with MELAS, independent of whether patients were at the acute and subacute phase or at the interictal phase. Although a few studies have provided evidence of impaired oxidative metabolism in the brains of MELAS patients, our study recruited a larger sample of patients with MELAS and demonstrated the change of cerebral OEF using MRI. Moreover, our study suggested a different degree of impairment of oxidative metabolism at various phases in the brains of MELAS patients. 

Our results of decreased cerebral OEF in the brains of MELAS are consistent with previous studies. Lindroos et al. performed a PET study in 14 patients, and showed a 26% decrease in the cerebral metabolic rate for oxygen in the gray matter and white matter, and a global decrease in the OEF regardless of the presence or absence of cerebral symptoms. The most striking finding of that study was that oxygen consumption was decreased in the brains of patients, including areas where no signs of disease were present [4]. The decrease of cerebral OEF at the interictal phase in our study confirmed those findings. COX II deficiency associated with MELAS results in deficient transfer of electrons through the redox group to oxygen, and hence decreases utilization of oxygen in the tissue. MR OEF imaging measures the concentration of deoxyhemoglobin in the tissue and reflects the efficiency of oxygen consumption. Our results indicate impairment of mitochondrial function of brain tissue even in the steady state of MELAS.

The underlying mechanism of stroke-like episodes remains controversial. The onset of stroke-like episodes is the leading cause of brain damage in MELAS. Although various hypotheses have been proposed, including a ischemic vascular mechanism [11], a generalized cytopathic mechanism [12], and a non-ischemic neurovascular cellular mechanism [13,14] none have been confirmed. Our study, together with previous findings, suggested a new possibility involving suppression of mitochondrial function in the genesis of stroke-like episodes. Our study showed that brains at the acute and subacute phase, especially for stroke-like lesions, may display marked decreases in OEF. Satoshi Takahashi et al. performed a PET study at 16 days and 35 days after a stroke-like episode, and found that OEF had decreased generally in the brain, particularly in the affected area at 16 days after the onset compared with 35 days [5]. These findings suggest that the mitochondrial function further deteriorated at the acute and subacute stages of MELAS. During the interictal period, brain tissue cells can maintain their functions, despite low level of ATP production. The rate of oxygen consumption should not change at the acute phase if the mitochondria work as usual. An increase in energy demand may impose pressure on the mitochondrial function, but would not cause the decrease in oxygen consumption. A recent study suggested the discrepancy between the decreased technetium-99m hexamethylpropyleneamine oxime uptake and increased rCBF at the hyperacute stage in MELAS, which might be caused by mitochondrial dysfunction [15]. 

**Figure 4:**
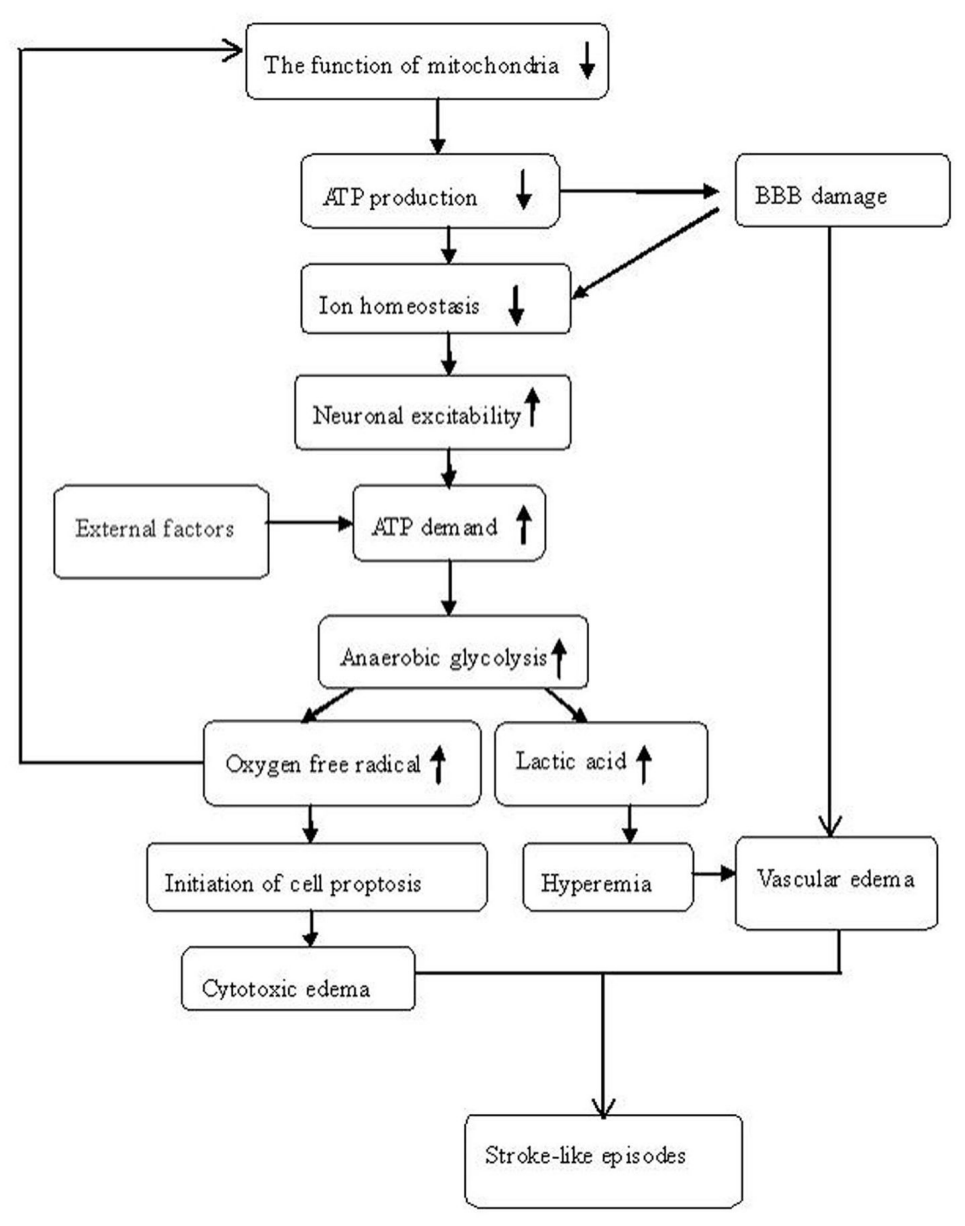
The mechanism of stroke-like episodes.

There are multiple factors that may cause the inhibition of mitochondrial function. Stable membrane potential is fundamental to the maintenance of mitochondrial function. A defect in oxidative phosphorylation may destabilize the membrane potential, resulting in alterations in ion homeostasis of nerve cells, such as neuronal calcium homeostasis [16]. Changes in the blood-brain barrier (BBB) may also inhibit mitochondrial function [17]. The extracellular ion concentration is tightly regulated by the BBB, and changes in extracellular ion concentration may depolarize adjacent neurons in the cortex leading to repetitive firing in a localized brain region, initiating a cascade of stroke-like events. Impaired energy metabolism in the capillary endothelium may also cause changes in extracellular ion homeostasis and vasogenic edema. In addition to these mechanisms, we propose that increased reactive oxygen species (ROS) may play a critical role. Mitochondria are a major source of ROS in human cells, and the production or accumulation of ROS was reported to be increased in patients with MELAS [18,19]. Overproduction of ROS from dysfunctional mitochondria may directly cause oxidative damage to cellular macromolecules such as lipids, proteins, and nucleic acids. Accumulation of ROS also destroys mitochondrial function and further deceases ATP synthesis. The progressive imbalance between ATP supply and demand leads to increased anaerobic glycolysis and increased ROS formation, which results in a vicious cycle. When the intrinsic pathway of programmed cell death is initiated, cytotoxic edema can be observed in the brain. The increased CBF in the lesion region is considered to be the result of increased anaerobic glycolysis [20] and some studies reported restricted diffusion reflected cytotoxic edema in the lesion [21-23]. Figure 4 denotes our hypothesis of the genesis of stroke-like episodes.

Further studies are required to support our hypothesis. Though it is inferred from the clinical course that the severity of MELAS is not equal among the phases, with the acute phase being most prominent, the degree of defective oxidative phosphorylation may be heterogenous across the brain, and there may be some subclinical lesions, we need to clarify the relationship between the severity of MELAS and OEF reduction more thoroughly. Moreover, the progressive and fluctuating course of MELAS requires serial imaging follow-up. We have reported serial OEF measurement throughout the stroke-like episodes in two cases [24], which showed a prominent OEF decrease at the onset of episodes and subsequent increase of OEF. Further data are required to examine the dynamic change of metabolism during the stroke-like episodes.

In conclusion, we obtained oxidative metabolism data in patients with MELAS by using MRI. Our study showed decreased utilization of oxygen in the brains of MELAS patients during the interictal period. When the stroke-like episodes attack, the mitochondrial abnormalities were more prominent, especially in the lesions, suggesting that mitochondrial dysfunction was exacerbated during the episode. With the detailed anatomical information available on MRI and the widely accessible MR scanners, the ability to quantitatively estimate OEF *in vivo* is likely to further our understanding of the pathophysiology and prognosis of MELAS. 
